# Additional Sex Combs-like Family Associated with Epigenetic Regulation

**DOI:** 10.3390/ijms25105119

**Published:** 2024-05-08

**Authors:** Nackhyoung Kim, Sukyoung Byun, Soo-Jong Um

**Affiliations:** Department of Integrative Bioscience and Biotechnology, Sejong University, 209 Neungdong-ro, Gwangjin-Gu, Seoul 05006, Republic of Korea; 22trul@gmail.com (N.K.);

**Keywords:** ASXL, epigenetics, mechanism, chromatin, regulation, transcription

## Abstract

The additional sex combs-like (ASXL) family, a mammalian homolog of the *additional sex combs* (*Asx*) of *Drosophila*, has been implicated in transcriptional regulation via chromatin modifications. Abnormal expression of ASXL family genes leads to myelodysplastic syndromes and various types of leukemia. De novo mutation of these genes also causes developmental disorders. Genes in this family and their neighbor genes are evolutionary conserved in humans and mice. This review provides a comprehensive summary of epigenetic regulations associated with ASXL family genes. Their expression is commonly regulated by DNA methylation at CpG islands preceding transcription starting sites. Their proteins primarily engage in histone tail modifications through interactions with chromatin regulators (PRC2, TrxG, PR-DUB, SRC1, HP1α, and BET proteins) and with transcription factors, including nuclear hormone receptors (RAR, PPAR, ER, and LXR). Histone modifications associated with these factors include histone H3K9 acetylation and methylation, H3K4 methylation, H3K27 methylation, and H2AK119 deubiquitination. Recently, non-coding RNAs have been identified following mutations in the ASXL1 or ASXL3 gene, along with circular ASXLs and microRNAs that regulate ASXL1 expression. The diverse epigenetic regulations linked to ASXL family genes collectively contribute to tumor suppression and developmental processes. Our understanding of ASXL-regulated epigenetics may provide insights into the development of therapeutic epigenetic drugs.

## 1. Introduction

Epigenetic regulation is a dynamic process that enables the activation or repression of genes at the transcriptional level in response to external stimuli, developmental cues, and cellular demands. It plays a particularly important role in development, cellular homeostasis, and cancer. The molecular mechanism underlying transcription involves the binding of transcription factors to specific DNA sequences for the regulation of target genes [1]. However, transcription factors typically do not act alone; they require the assistance of transcriptional coregulators to precisely manage gene expression. Coregulators, a diverse group of proteins, interact directly or indirectly with transcription factors. Participating in epigenetic regulation, coregulators modify the chromatin structure by inducing biochemical changes, such as methylation, acetylation, and other modifications. Considering their essential role in regulating the expression of genes controlling metabolism and cell fate, dysregulation of coregulators has been implicated in various defects, including metabolic disorders and malignancy [2,3].

The *additional sex combs* (*Asx*) gene in *Drosophila* acts as a transcriptional coregulator during embryonic development [4,5,6]. The mammalian homolog, known as additional sex combs-like (*ASXL*) gene (including *ASXL1*, *ASXL2*, and *ASXL3*), shares functional similarities [7,8,9]. In *Drosophila*, *Asx* participates in both transcriptional repression and activation through genetic interactions with the polycomb-repressive complex 2 (PRC2) or trithorax group (TrxG). It is currently unclear whether the mammalian ASXL family functions in transcriptional regulation similar to *Drosophila* Asx. This function may be influenced by promoter context, extracellular signals, or targeted transcription factors associated with epigenetic modifications. *ASXL1* mutations are associated with disorders such as Bohring–Opitz syndrome (BOS), acute myeloid leukemia (AML), and embryonic developmental defects [10,11,12,13,14]. While the molecular biology of *ASXL1* has been partially clarified, certain epigenetic mechanisms remain elusive. This review provides insight into the epigenetic regulation governed by the *ASXL* family.

## 2. Genetic and Structural Conservation

**Figure 1:**
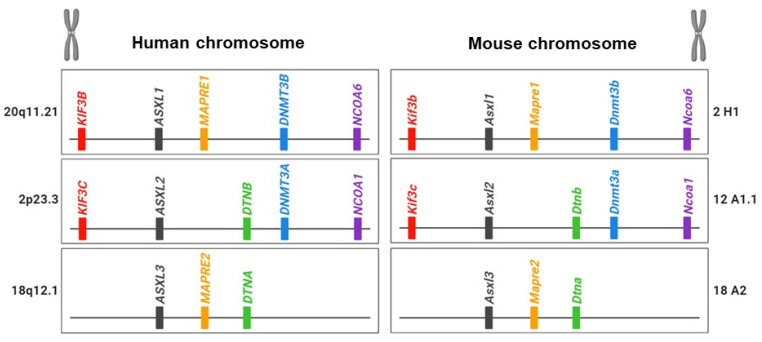
Illustration of the *ASXL* family and their neighbor genes in human and mouse genomes.

The chromosomal loci of *ASXL* family genes vary among family members and across species: for example, there is human hASXL1 at 20q11, hASXL2 at 2p23.3, and hASXL3 at 18q12.1 but murine mAsxl1 at 2H1, mAsxl2 at 12A1.1, and mAsxl3 at 18A2 (Figure 1). 

Unlike other species, *Drosophila* lacks a familial gene for Asx. The increased number of *ASXL* homologous genes may stem from gene duplication, leading to the evolution of new biological functions or divergence in the DNA sequence from the original gene [15]. Genetic evidence suggests that the *ASXL* family underwent gene duplication during evolution. Sequence comparisons of *ASXL* family genes suggest that *ASXL2* and *ASXL3* originated from a duplication of the ancestral *ASXL1* gene during early mammalian evolution. In addition to the sequence similarities within the *ASXL* family, there is evidence of evolutionary conservation of neighboring genes around *ASXL* family members in both humans and mice (Figure 1) [15]. The *KIF3B* gene is located upstream of *ASXL1*, whereas *KIF3C* is positioned upstream of *ASXL2*. *DNMT3B/NCOA6* and *DNMT3A/NCOA1*, common neighbor genes, are located downstream of *ASXL1* and *ASXL2*, respectively. *DTNB* and *DTNA* genes are shared downstream genes for *ASXL2* and *ASXL3*. *NOL4L* and *NOL4* genes are downstream of *ASXL1* and *ASXL3*. The presence of related neighboring genes around the *ASXL* family suggests that the evolutionary conservation of paralogous *ASXL* genes plays crucial roles in functional diversification and contributes to essential regulatory elements in biological processes, reflecting the evolutionary history of the genome.

**Figure 2:**
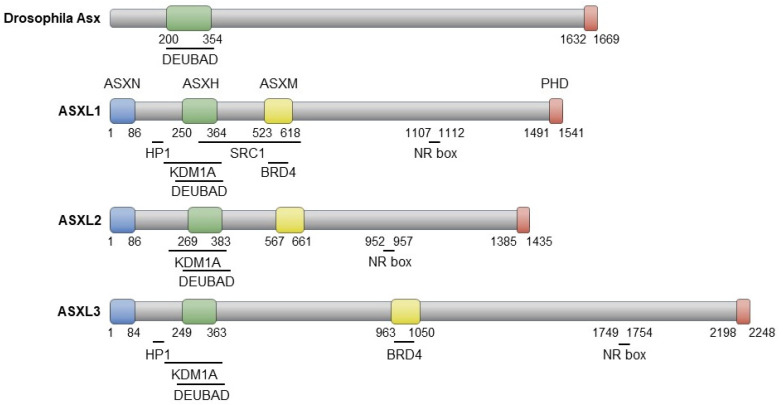
Schematic representation of Asx and ASXL proteins, highlighting their interaction domains with partner proteins.

The three proteins encoded by *ASXL* family genes exhibit evolutionarily conserved domains similar to Asx, including the ASX N-terminal domain (ASXN), the ASX homologous domain (ASXH), and the plant homeodomain (PHD) (Figure 2). While *Drosophila* Asx consists of 1669 amino acids (aa), its human homologs have varying lengths: 1541 aa (ASXL1), 1435 aa (ASXL2), and 2248 aa (ASXL3) [16]. The N-terminal ASXN domain contains the HARE-HTH motif, absent in Asx, and is predicted to mediate DNA binding [17]. The ASXH domain, highly conserved in both Asx and ASXL members, includes a DEUBAD domain that interacts with and activates BAP1 (Calypso in *Drosophila*) to remove ubiquitin from the monoubiquitinated histone H2A at lysine 119 (H2AK119ub) [18,19,20]. The C-terminal PHD finger is implicated in preferential binding to dimethylated histone 3 lysine 4, H3K4me2 [21]. The nuclear receptor (NR) box, responsible for nuclear hormone receptor-mediated transcriptional regulation, is conserved in the ASXL family [21,22,23,24,25]. ASXL1 and ASXL3 share the heterochromatin protein 1 (HP1)-binding motif, which is absent in ASXL2 [23]. The ASXH domain in the ASXL family is responsible for interaction with the histone demethylase KDM1A (LSD1) [21,25]. In addition, an interaction between the ASXM domain of ASXL1/3 and BRD4, a member of the bromodomain and extraterminal (BET) proteins, has recently been reported [26,27].

## 3. Tumor Suppression

Mutations in ASXL1, a candidate tumor suppressor gene, are frequently observed in myeloid malignancies, such as acute myeloid leukemia and myelodysplastic syndrome (MDS), which are often associated with a poor prognosis [11,12,28,29,30]. The predominant *ASXL1* mutations involve frameshift or nonsense mutations in exon 12, causing the expression of truncated forms of ASXL1 [31,32,33]. These mutations typically lead to a loss of protein, but in some instances, they result in the production of truncated proteins with gain-of-function or dominant-negative features [32,34,35,36]. Numerous studies have demonstrated that truncated *ASXL1* mutants, including the ASXL1 fragment containing amino acids 1–587, promote myeloid transformation by forming a stable polycomb-repressive deubiquitinase (PR-DUB) complex with BAP1, enhancing BAP1 deubiquitinase (DUB) activity [37,38,39]. This mutant also interacts with BRD4 and activates the transcription of genes involved in myeloid malignancies [27,40]. However, the precise mechanism by which ASXL1 mutations acquire a dominant-negative function needs further investigation. Several mouse models have been developed to investigate the impact of changes in ASXL1 on hematopoiesis and myeloid transformation [13,41,42,43,44]. ASXL1 is also considered a tumor suppressor in other types of cancers, including that of the prostate, colorectal, and lung [45,46,47]. Database analysis of circular RNAs in bladder cancer has shown that circASXL1 is highly expressed in bladder cancer tissues and correlated with overall survival [48].

ASXL2 is believed to have overlapping or redundant functions with ASXL1 due to their similarity in protein domains, expression patterns, and neighboring gene sets at their genomic loci. However, they differ in certain aspects. Unlike the high mutation rate in exon 12 of ASXL1, ASXL2 mutations in myeloid malignancies are found at exons 11 and 12 [49]. Notably, ASXL2 mutations occur frequently in acute myeloid leukemia with t(8;21)/RUNX1-RUNX1T1 and less frequently in other myeloid malignancies, indicating mutual exclusivity with *ASXL1* mutations [49,50]. Mouse studies have suggested that Asxl2 loss dysregulates the self-renewal of hematopoietic stem cells and accelerates leukemogenesis driven by AML1-ETO, indicating distinct effects from *Asxl1* deletion [51]. Another study demonstrated that *Asxl2* deletion leads to a myelodysplastic syndrome-like disease and increases the self-renewal of hematopoietic stem cells [52]. The mutual exclusivity between *ASXL1* and *ASXL2* mutations remains unclear. Similar to ASXL1, ASXL2 forms a stable and distinct PR-DUB complex with BAP1, promoting ubiquitin removal from histone H2A. However, unlike ASXL1, ASXL2 is stabilized by BAP1 [53,54], indicating the existence of additional regulatory mechanisms. ASXL2 is also associated with solid tumors. Elevated ASXL2 expression is linked to poor survival and is correlated with the prognosis of breast, colorectal, and pancreatic cancers [21,55,56], whereas its downregulation is associated with hepatocellular carcinoma [57]. In mice, ASXL2 loss leads to myeloid leukemia, suggesting a dual role in tumorigenesis.

The relationship between ASXL3 and tumor development is unclear, as it is rarely mutated and not as closely associated with leukemia as ASXL1 and ASXL2 [58,59]. However, recent studies have found its potential role in certain cancers. A study demonstrated that ASXL3 forms an oncogenic axis with BRD4 and BAP1, activating ASCL1/MYCL/E2F signaling in small cell lung cancer [26]. Despite its limited involvement in tumorigenesis, changes in ASXL3 are implicated in developmental defects, congenital heart disease, and Bainbridge–Ropers syndrome (BRS) [60,61,62], which shares similarities with BOS, caused by autosomal truncations in ASXL1 [63,64].

## 4. Developmental Roles

In addition to their roles in myeloid malignancies, deletion studies in mice have shown that both Asxl1 and Asxl2 regulate hematopoiesis [41,42,43,44,51,52], whereas the role of Asxl3 in hematopoiesis remains unexplored. De novo mutations in ASXL family members are associated with various developmental defects: ASXL1 mutations are linked to BOS, ASXL2 mutations to Shashi–Pena syndrome (SPS), and ASXL3 mutations to BRS [10,62,63,64,65,66]. ASXL1 mutations leading to BOS are characterized by distinctive facial features, cleft palates, intellectual disability, microcephaly, breathing problems, skeletal abnormalities, and eye defects [10,65,67]. However, the molecular mechanisms underlying the role of such mutations in causing BOS are not fully understood. In vitro studies have shown that Asxl1 ablation in embryonic stem cells from mice impairs neural differentiation [68]. ASXL1-deleted mouse models have defects in kidney podocyte development [69], embryonic fibroblast proliferation [70], and embryonic lung maturation [14]. Germline mutations of ASXL2 cause developmental syndromes, including SPS, characterized by the absence of slowed growth and microcephaly [66,71]. ASXL3 mutations leading to BRS are associated with intellectual disability, developmental delay, and speech and language difficulties. However, the underlying molecular mechanisms remain unclear [63,72,73]. This different phenotype suggests that the epigenetic mechanisms of ASXL1/3 and ASXL2 may differ. Notably, ASXL1 and ASXL2 have opposite roles in mediating adipogenesis and lipogenesis in vitro [23,24], with ASXL1 demonstrating similar effects to ASXL3 in repressing LXRα during lipogenesis [25].

## 5. Epigenetic Associations

### 5.1. CpG Islands and DNA Methylation

**Figure 3:**
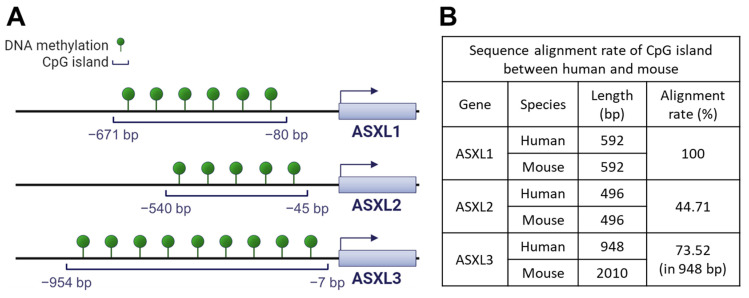
Conserved CpG islands of ASXL genes in human and mouse. (**A**) CpG islands in the three human ASXL genes. CpG island analysis in ASXL family genes was performed using the DBCAT web tool (http://dbcat.cgm.edu.tw/, accessed on 24 March 2023). (**B**) Alignment rate of CpG island of ASXL genes between human and mouse. The alignment rate was analyzed using the Multalin web tool (http://multalin.toulouse.inra.fr/multalin/, accessed on 25 March 2023).

In addition to their functional similarity, ASXL familial genes share a common genetic feature: CpG islands, evolutionarily conserved in both humans and mice, are located before the transcriptional starting sites of genes (Figure 3). Using hg18 as the reference genome for humans and mm9 for mice, the length of the CpG island for human ASXL1 and mouse Asxl1 genes is identical at 592 base pairs, with identical sequences (Figure 3).

These findings suggest the importance of *ASXL1* gene regulation, preserved throughout evolution. Both human ASXL2 and mouse Asxl2 have the same CpG island length (i.e., 496 bp). However, the alignment rate of the CpG island between humans and mice is only 44.71%. The CpG islands of ASXL3 differ in length between humans (948 bp) and mice (2010 bp), with a relatively high alignment ratio (73.52%), suggesting that the activity of *ASXL* genes may be regulated by DNA methylation at CpG islands. In line with this, the publicly available dataset GSE81680, generated by methylated DNA immunoprecipitation sequencing, provides evidence of DNA methylation around ASXL genes in murine embryonic stem cells [74]. Conversely, genome-wide DNA methylation profiles could be altered by *ASXL1* mutations [75,76]. The interaction between Asxl1 and Wtip during podocyte development suggests that Asxl1 regulates DNA methylation [69]. Wtip interacts with the transcription factor WT1, which in turn regulates DNA methylation by interacting with the TET2 enzyme [77]. However, whether Asxl1 or other family members participate in the regulation of DNA methylation via WT1 or TET2 has not been investigated. Considering the prognostic and therapeutic significance of *ASXL1*, *WT1*, and *TET2* mutations in myeloid leukemia, further investigations are needed to elucidate the mechanisms underlying the transcriptional regulation of *ASXL* genes through DNA methylation. 

### 5.2. Histone Modifications

**Figure 4:**
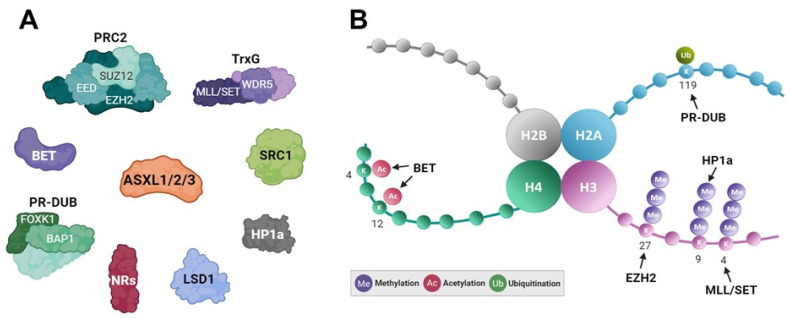
Schematic representation of transcriptional mechanism related to the ASXL family. (**A**) Epigenetic regulators interacting with the ASXL family. (**B**) Histone tails and their modifications by ASXL-associated epigenetic modifiers and readers (BET and HP1a).

Early studies on *Drosophila* Asx revealed its dual role as a member of the “enhancers of trithorax and polycomb” (ETP) group, influencing epigenetic processes through differential histone modifications. It represses or activates transcription by modulating the trimethylation levels of histone H3 lysine 27 (H3K27me3) or histone H3 lysine 4 (H3K4me3) through direct interaction with histone methyltransferases Enhancer of zeste E(z), a member of the polycomb group, or by Trx, a member of the Trithorax group, respectively [6,78]. In mammalian systems, ASXL family members interact with various histone modifiers, including PRC2, TrxG, BAP1 deubiquitinase, NCOA1 (SRC1), HP1α, histone demethylase KDM1A (LSD1), and BRD4 (Figure 4A). However, their functions and underlying mechanisms in transcriptional regulation are unclear. The role of the ASXL family in regulating gene expression through interactions with PRC2 has been extensively investigated [70,79,80]. Enhancer of zeste homolog 2 (EZH2), a key component of PRC2, acts as a histone methyltransferase, catalyzing the trimethylation of H3K27, leading to transcriptional repression (Figure 4B). ASXL1 participates in transcriptional repression by interacting with EZH2 [42,70,79]. ASXL2 also mediates chromatin recruitment of EZH2 and increases H3K27me3 levels [80]. Compared to ASXL1 or ASXL2, the function of PRC2-associated ASXL3 remains unexplored. In particular, considering the frequent mutations of ASXL1 in various types of leukemia, the synergistic role of ASXL2 and PRC2 complex in leukemia development and hematopoiesis has been explored [80]. 

Recent studies have demonstrated that lysine demethylase 6B (KDM6B), which demethylates H3K27me3, is elevated in ASXL1-mutant leukemic cells [81]. This upregulation enhances the expression of leukemogenic genes and contributes to myeloid transformation. The effects of KDM6B have been validated through the heterozygous deletion of Kdm6b in Asxl1Y588XTg mice. The histone modification H3K4me3 is a crucial epigenetic marker of active gene expression [82,83]. TrxG proteins include the histone lysine methyltransferases MLL and SET1, which regulate H3K4me3 by adding methyl groups to histone H3 at lysine 4 and modulate lineage commitment during differentiation [84,85,86]. The bivalent histone code, involving both H3K4me3 and H3K27me3, is essential for maintaining gene expression patterns during differentiation and development [87,88]. Unlike the dual role observed in the *Drosophila* Asx protein, the mammalian ASXL family shows diverse functions, functioning as a coactivator or corepressor, depending on specific isotypes and promoter contexts. ASXL family members exhibit distinct roles in modulating the transcriptional activity of NRs by influencing various histone modifications (Table 1) [21,22,23,24,25]. In particular, ASXL1 and ASXL3 act as corepressors for certain NRs (PPARα and LXRα) by interacting with HP1α, resulting in an increase in the repressive histone mark H3K9me3. Conversely, ASXL1 acts as a coactivator for retinoic acid receptor α (RARα) by interacting with SRC1, a histone acetyltransferase, leading to the accumulation of acetylated H3K9. ASXL2 acts as a coactivator for estrogen receptor α (ERα) by upregulating the active histone mark H3K4me3 and downregulating the repressive marks H3K9me2 and H3K27me3. Despite their similar structure and domain arrangements, the precise mechanism underlying the bivalent roles of the ASXL members in modulating H3K27me3 and H3K4me3 remain elusive.

In addition to the bivalent epigenetic mechanism involving H3K27me3 and H3K4me3, the ASXL family plays a crucial role in regulating histone H2A (H2AK119ub) ubiquitination, an essential epigenetic process during cellular differentiation, organ development, and disease pathology [89,90]. H2AK119ub is catalyzed by PRC1 and subsequently removed by the polycomb-repressive deubiquitinase (PR-DUB) complex, including BAP1 and ASXL1 in both *Drosophila* and mammals (Figure 4B) [18,19,20]. EZH2, a component of core PRC2, is the key enzyme responsible for catalyzing H3K27me3. Subsequently, PRC1 recognizes H3K27me3 through CBX, leading to H2A ubiquitination via RING1B for gene repression [91]. Truncated ASXL1 mutants promote myeloid transformation by creating a potent PR-DUB complex with BAP1 [37,38,39]. ASXL2 interacts with the C-terminal domain of BAP1 and enhances PR-DUB activity. In cancer cells expressing a BAP1 mutant defective in ASXL2 binding, PR-DUB activity is disrupted, suggesting that BAP1 C-terminal domain mutations may contribute to cancer development [53]. Intriguingly, ASXL3, similar to ASXL1 and ASXL2, forms a PR-DUB complex with BAP1 but also exclusively interacts with BRD4, which binds to acetylated histones via its bromodomains in small cell lung carcinoma [26]. The intricate epigenetic coordination between H3K27me3 catalyzation and H2AK119ub elimination by ASXL family members provides insights into the regulation mediated by ASXL proteins.

Recent studies have revealed that ASXL proteins are physically and functionally linked to histone acetylation (Figure 4B) [26,27,40]. The BET protein family, acting as an epigenetic reader of acetylation for histones H3 and H4, is associated with the RNA polymerase II complex to activate transcription [92,93]. During leukemogenesis, truncated ASXL1 acts as a gain-of-function mutant through interaction with BRD4, a BET protein [26,40]. Although previous studies have mainly focused on the physical interaction between ASXL1–3 and BRD4 and the biological significance of truncated ASXL1 in hematological malignancies, the epigenetic role of this interaction in regulating target genes and histone acetylation needs further exploration. In small cell lung cancer patients, BRD4 interacts with ASXL3 but not ASXL1 or ASXL2 [27]. The PR-DUB.3 complex shares common target genes with BRD4 through its interaction with ASXL3. Although the binding of PR-DUB.3 and BRD4 to target genes has been validated by chromatin immunoprecipitation followed by sequencing, the precise epigenetic mechanism driving the oncogenic function of the ASXL3 complex remains unclear. Moreover, it is essential to explore the physical interaction between ASXL1–3 and other BET proteins, such as BRD2, BRD3, and BRDT, and to investigate their biological and epigenetic roles during tumorigenesis and developmental processes. Understanding these mechanisms could lead to the development of epigenetic drugs, such as BET inhibitors, for cancer therapy.

### 5.3. Non-Coding RNAs (ncRNAs)

NcRNAs constitute a diverse group of RNAs that perform various biological functions, independent of translation [94,95]. They can be categorized based on their length, shape, or function. MicroRNAs (miRNAs), typically consisting of 21–23 nucleotides and forming short hairpins before maturation, serve as epigenetic regulators by interacting with target mRNAs and suppressing their expression. Circular RNAs (circRNAs), characterized by a single-stranded RNA with a covalently closed continuous loop, can be generated through the RNA splicing process. They play an indirect role in epigenetic regulation by acting as miRNA sponges, where miRNAs are sequestered against the complementary region of circRNA, resulting in enhanced expression of the corresponding miRNA target genes. Furthermore, they serve as sequestering agents for RNA-binding proteins and transcription factors. Their interactions with transcriptional regulators can impact the enzymatic activities of epigenetic modifiers. Although numerous approaches have been used to investigate the role of ASXLs in epigenetics, the biological relationship between the ASXL family and ncRNAs has not been fully explored. Notably, *ASXL1* gene mutations with C-terminal truncations lead to elevated miR-125a expression by disrupting the EZH2-mediated methylation of H3K27, resulting in a myelodysplastic syndrome-like disease in mice [42]. The increased miR-125a level downregulates *Clec5a* expression, which is associated with normal myeloid differentiation. Moreover, during DMSO-induced myocardial differentiation of P19 cells, ASXL3 knockdown induces differential expression of various miRNAs linked to the PI3K-Akt, MAP kinase, and Rap1 signaling pathways, as well as heart development [96]. 

Unlike the ncRNAs regulated by ASXL proteins, certain circRNAs are transcribed at the gene loci of the ASXL family. For instance, circASXL1 (circBase ID: hsa_circ_0001136), initially identified through the circular RNA database related to bladder cancer, is associated with tumor grade and shorter overall survival [48]. In colorectal cancer (CRC) progression [97], circASXL1 induces GRIK3 expression by sponging miR-1205, thereby promoting tumor growth. Additional circASXL1 variants have been identified through RNA sequencing in leukemic cells, with one reported to bind directly to BAP1, inhibiting the deubiquitinase activity of the PR-DUB complex [98]. However, the mechanism by which circASXL1 interferes with BAP1 activity and whether it affects ASXL1 expression through a feedback loop remain to be determined. ASXL1 is also susceptible to downregulation by specific ncRNAs. For instance, circ-ITGA7, downregulated in CRC cells, suppresses CRC proliferation by sponging miR-3187–3p, which potentially targets ASXL1–5′UTR, resulting in the silencing of ASXL1 expression [99]. On the other hand, LINC00586, a long ncRNA, exhibits high expression in CRC and promotes tumorigenesis by recruiting LSD1 into the ASXL1 promoter, causing ASXL1 downregulation [100]. In addition to cancers, circASXLs have been implicated in other diseases. For instance, the role of circAsxl2 in neuronal injury has been demonstrated in the neuronal cells of mice [101], revealing that it is upregulated in cells subjected to oxygen-glucose deprivation/reperfusion treatment, leading to increased Foxo3 expression through sponging miR-130b-5p. However, the biological function of circASXL3 remains unexplored. Further exploration on the roles of ncRNAs linked to the ASXL family may facilitate biomarker identification and advancements in epigenetic therapy.

## 6. Conclusions and Future Perspectives

Despite the first documentation of the biological function of the *Drosophila* Asx gene in 1986, our understanding of the various mechanisms utilized by the chromatin factor Asx or the ASXL family in mammals to regulate ASXL-related physiological processes remains incomplete, particularly at the epigenetic level. Most studies have investigated the mutation sites and their role in cancer. For example, the role of different truncated ASXL1 (aa 1–587, aa 1–635, aa 1–643, aa 1–646) in hematopoietic stem cells has been investigated to understand the ASXL association in cancer progression [37,38,39,40,42]. However, the development of therapeutic strategies based on the molecular mechanisms of ASXL mutants is still lacking. De novo mutations in ASXL members can cause severe developmental disorders, but our understanding of the underlying molecular mechanisms is currently limited. The tissue-specific functions of *ASXL* genes can be determined through the conditional deletion of these genes in mice. Primary cells, including stem cells derived from ASXL-deleted mice, offer a valuable resource to investigate the molecular mechanisms underlying ASXL-associated physiological processes. Recently, several notable advancements have been made in the knowledge of epigenetics, based on molecular biological techniques and innovative bioinformatic technology. For a comprehensive understanding of the dynamic regulation of target genes at chromatin, linked to DNA methylation and histone modification, further identification of transcription factors beyond NRs [21,22,23,24,25] or the FOXK family [102,103] may extend our understanding of the chromatin localization of the ASXL family and their biological importance at the transcriptional level. In addition, genome-wide studies, encompassing ChIP sequencing, ATAC sequencing, and chromosome conformation capture (3C) technology coupled with high-throughput sequencing, are necessary to unveil the role of ASXL proteins in orchestrating chromatin rearrangement and three-dimensional genome organization at specific genomic loci. To investigate genomic interactions, an initial approach would be to examine the physical interaction between ASXL proteins and the CCCTC-binding factor/cohesin complex, a regulator of high-order chromatin organization. Truncated ASXL1 mutants, specifically the ASXL1 fragment containing amino acids 1–587, drive myeloid transformation by forming a stable PR-DUB complex with BAP1, thereby enhancing BAP1 deubiquitinase activity [37,38,39]. This mutant also interacts with the BET protein BRD4, activating the transcription of genes involved in myeloid malignancies [26,40]. In contrast to the oncogenic function of truncated ASXL1 mutants, the mechanism underlying the tumor-suppressing role of full-length ASXL1 requires exploration across various types of cancers, including leukemia. Peptide pull-down and ChIP assays can be employed to demonstrate the interaction between ASXL1 and BET proteins (BRD2–4 and BRDT) disrupting BET association with acetylated chromatin (at lysine 5 and 12 of histone 4 or lysine 14 of histone 3) through its bromodomain, leading to downregulation of target oncogenes such as MYC and BCL2. Subsequent studies should involve other ASXL family members, ASXL2 and ASXL3, to ascertain whether their functions are redundant or distinct in cancers and developmental defects. The crucial involvement of ASXL family genes in cancer and development suggests that exploring novel epigenetic drugs targeting their underlying molecular mechanisms could present a promising avenue for therapeutic development.

## Figures and Tables

**Table 1:** Histone modifications associated with ASXL-mediated nuclear receptor regulation.

ASXL	NR *	Interactions	Transcription	Histone Marks **	Reference
ASXL1	RARα	SRC1	Activation	H3K9ac ↑	[32]
	PPARγ	HP1α	Repression	H3K9me3 ↑	[33]
	LXRα	ND *	Repression	ND	[34]
ASXL2	ERα	LSD1, UTX, MLL2	Activation	H3K9me2 ↓, H3K27me3 ↓, H3K4me3 ↑	[30]
	PPARγ,	MLL1	Activation	H3K9ac ↑, H3K4me3 ↑	[33]
	LXRα	ND	Activation	ND	[34]
ASXL3	LXRα	LSD1, HP1α	Repression	ND	[35]

* NR, nuclear receptor. **: ↑, increased; ND, not determined; ↓, decreased.

## Data Availability

Not applicable.
